# Antimicrobial Resistance and Genomic Epidemiology of *tet*(X4)-Bearing Bacteria of Pork Origin in Jiangsu, China

**DOI:** 10.3390/genes14010036

**Published:** 2022-12-22

**Authors:** Yuhan Li, Yan Li, Kefan Bu, Mianzhi Wang, Zhiqiang Wang, Ruichao Li

**Affiliations:** 1 Jiangsu Co-Innovation Center for Prevention and Control of Important Animal Infectious Diseases and Zoonoses, College of Veterinary Medicine, Yangzhou University, Yangzhou 225009, China; 2 Institute of Comparative Medicine, Yangzhou University, Yangzhou 225009, China; 3 Joint International Research Laboratory of Agriculture and Agri-Product Safety, The Ministry of Education of China, Yangzhou University, Yangzhou 225009, China

**Keywords:** *tet*(X4), plasmids, food safety, genomics

## Abstract

The emergence of tigecycline-resistant bacteria in agri-food chains poses a public health concern. Recently, plasmid-mediated *tet*(X4) was found to be resistant to tigecycline. However, genome differences between *tet*(X4)-positive *Escherichia coli* of human and pork origins are still under-investigated. In this study, 53 pork samples were collected from markets in Jiangsu, China, and 23 *tet*(X4)-positive isolates were identified and shown to confer resistance to multiple antibiotics, including tigecycline. *tet*(X4)-positive isolates were mainly distributed in *E. coli* (n = 22), followed by *Klebsiella pneumoniae* (n = 1). More than half of the *tet*(X4) genes were able to be successfully transferred into *E. coli* C600. We downloaded all *tet*(X4)-positive *E. coli* isolates from humans and pork found in China from the NCBI database. A total of 42 known STs were identified, of which ST10 was the dominant ST. The number of ARGs and plasmid replicons carried by *E. coli* of human origin were not significantly different from those carried by *E. coli* of pork origin. However, the numbers of insertion sequences and virulence genes carried by *E. coli* of human origin were significantly higher than those carried by *E. coli* of pork origin. In addition to *E. coli*, we analyzed all 23 *tet*(X4)-positive *K. pneumoniae* strains currently reported. We found that these *tet*(X4)-positive *K. pneumoniae* were mainly distributed in China and had no dominant STs. This study systematically investigated the *tet*(X4)-positive isolates, emphasizing the importance of the continuous surveillance of *tet*(X4) in pork.

## 1. Introduction

In recent years, multidrug-resistant (MDR) Gram-negative bacteria have posed a serious threat to public health [1,2]. Because of its broad-spectrum antibacterial activity, tigecycline is considered the last resort in the clinical treatment of infection caused by MDR bacteria [3,4]. Tigecycline belongs to a class of drugs called glycylcyclines. Similar to tetracycline, it can reversibly bind to the 30 S subunit of the ribosome, interfering with amino acid translation and inhibiting bacterial growth [5,6]. However, He et al. discovered the plasmid-mediated mobile tigecycline resistance genes *tet*(X3) and *tet*(X4) in Enterobacteriaceae and *Acinetobacter* in 2019 [7]. The *tet*(X4) gene often possesses complex genetic environments and is distributed in plasmids of multiple plasmid replicon types [8]. Notably, previous studies have shown that pork is an important reservoir of *tet*(X4) [9,10]. However, studies on the genomic epidemiology of *tet*(X4) in pork are still lacking.

The *tet*(X4) gene has been identified in a variety of Enterobacteriaceae, such as *E. coli*, *K. pneumoniae*, *Aeromonas caviae* and *Escherichia fergusonii* [10,11]. However, the vast majority of reported *tet*(X4) are distributed in *E. coli*. Furthermore, the presence of *tet*(X4) usually does not result in a significant fitness cost to *E. coli*, which further exacerbates the spread of *tet*(X4) in *E. coli* [10]. In addition to *E. coli*, the *tet*(X4) gene was sporadically detected in *K. pneumoniae* of different sources, including human sources and pork samples [10,12]. In this study, we analyzed the emerging *tet*(X4)-positive isolates isolated from pork samples in Yangzhou, China, in 2021. Meanwhile, we also compared the genomic differences of all reported *tet*(X4)-positive *E. coli* from human and pork sources in China using genomics methods, providing a genomic landscape of *tet*(X4)-positive isolates from various sources.

## 2. Materials and Methods

### 2.1. Bacterial Isolates

The 53 pork samples were randomly collected from markets in Yangzhou, China, in May 2021. Tigecycline-resistant isolates were selected on MacConkey agar plates with tigecycline (4 mg/L). 16S rRNA gene sequencing was used to perform bacterial species identifications of purified isolates. The *tet*(X4) gene was determined by PCR with reported primers [7]. 

### 2.2. Antimicrobial Susceptibility Testing

The minimum inhibitory concentrations (MICs) of *tet*(X4)-positive isolate strains were conducted against nine antibiotics and antimicrobials, including chloramphenicol, ciprofloxacin, meropenem, florfenicol, streptomycin, colistin, cefoperazone, tigecycline and tetracycline. *E. coli* ATCC 25922 was used as the quality control strain. The resistance breakpoint was interpreted according to the EUCAST criteria (>0.5 mg/L, V12.0) for tigecycline and CLSI guidelines for other antimicrobials [13]. 

### 2.3. Conjugation Experiments

The assessment of the transferability of the *tet*(X4) gene was conducted by conjugation experiments using *tet*(X4)-positive isolates as the donor strains and rifampicin-resistant *E. coli* C600 (RifR) as the recipient strain (1:1) at 37 °C [14]. The transconjugants were recovered on LB agar plates containing rifampicin (300 mg/L) and tigecycline (4 mg/L). PCR was used to further confirm the transconjugants. The plasmid replicon types carried in the original isolates and corresponding transconjugants were identified by PCR (Table S1).

### 2.4. Whole Genome Sequencing

According to the results of bacterial species identification and resistance phenotypes, six representative isolates were selected for WGS. The genomes of tigecycline-resistant strains were extracted with the FastPure bacteria DNA isolation Minikit (Vazyme, China) and quantified by a Qubit 4 Fluorometer. The genomic DNA samples were sequenced using the Illumina Hiseq 2500 platform with a 2 × 150 bp paired-end library. The paired-end reads were de novo assembled using SPAdes version 3.14.0 with the default parameters. 

### 2.5. Bioinformatics Analysis

The assembled sequences were annotated through the RAST online server (https://rast.nmpdr.org/, accessed on 1 August 2022) automatically. ResFinder, PlasmidFinder and ISfinder with the default parameters were used to detect the antibiotic resistance genes (ARGs), plasmid replicon types and insertion sequences [15,16,17]. For *tet*(X4)-carrying *K. pneumoniae* that was only sequenced with short-read sequencing, the contigs acquired by de novo assembly were aligned with *tet*(X4)-positive circular plasmids carrying different replicons to obtain the *tet*(X4)-positive plasmid types [18]. Virulence genes were determined using ABRicate (https://github.com/tseemann/abricate, accessed on 1 August 2022) and Kleborate (https://github.com/katholt/Kleborate, accessed on 1 August 2022). The multi-locus sequence types (MLST) of all *tet*(X4)-positive isolates were assigned using the mlst software (https://github.com/tseemann/mlst, accessed on 1 August 2022). Phylogenetic trees of *E. coli* and *K. pneumoniae* were constructed using Roary and FastTree based on single nucleotide polymorphisms (SNPs) of core genomes [19,20]. The phylogeny analysis was visualized and retouched using iTOL (https://itol.embl.de, accessed on 18 August 2022). 

### 2.6. Data Availability

The sequences obtained in this paper have been deposited in the GenBank database under the BioProject number PRJNA900003.

## 3. Results

### 3.1. Characterization of tet(X4)-Bearing Isolates among Pork

A total of 23 tigecycline-resistant isolates were collected from 53 pork samples. The 16S rRNA gene analysis showed that they were all *E. coli* (95.65%), except one that belonged to *K. pneumoniae* (4.35%). Antimicrobial susceptibility testing showed that these isolates all belonged to MDR isolates. Except for tigecycline (8–128 mg/L), these isolates were also resistant to other antibiotics such as florfenicol, chloramphenicol, streptomycin and tetracycline. However, all these isolates were susceptible to colistin and meropenem (Table S2).

### 3.2. Transferability of the tet(X4) Gene

To evaluate the transferability of *tet*(X4) in these isolates, conjugation assays were performed for these *tet*(X4)-positive isolates with *E. coli* C600 as the recipient. The *tet*(X4) gene in 14 isolates, including 13 *E. coli* isolates and 1 *K. pneumoniae* isolate, was successfully transferred to C600. The results of plasmid replicon typing showed that the *tet*(X4) gene was mainly located on IncX1-IncHI2A hybrid plasmids (35.71 %), followed by IncX1 plasmids (21.43 %) (Table S3).

### 3.3. Phylogenetic Analysis of tet(X)-Positive E. coli

**Figure 1:**
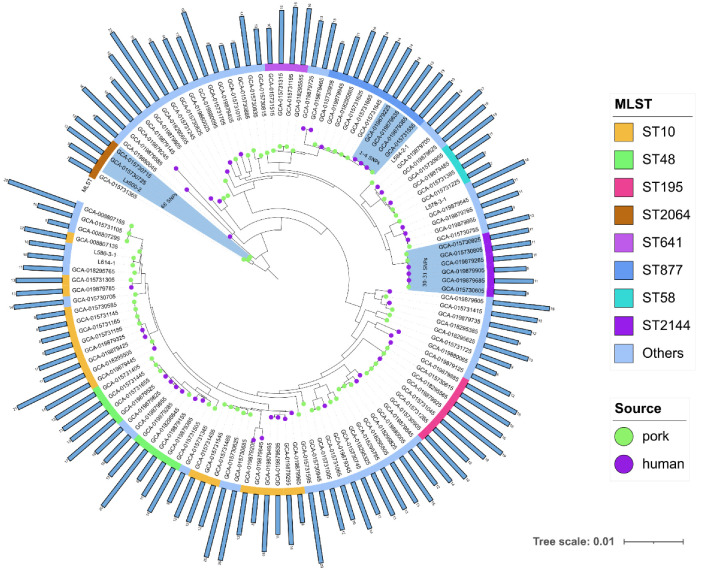
Phylogenetic analysis of 122 *tet*(X4)-positive *E. coli* isolates from pork and human samples. Blue-shaded areas represent strains with minor SNP differences. Histograms represent the number of resistance genes carried in the isolates.

To further investigate the evolutionary relationship of the *E. coli* isolated from pork samples, we downloaded all genomes of *tet*(X)-positive *E. coli* isolated from humans (n = 48) and pork (n = 69) in the NCBI database and constructed a phylogenetic tree based on SNPs of the core genomes (Figure 1, Table S4). We noted that some *tet*(X)-positive *E. coli* isolated from pork samples share high similarity (1–68 SNPs) with *tet*(X)-positive *E. coli* collected from a human source, and there is a possibility of clonal transmission. The MLST analysis showed that these *tet*(X4)-positive *E. coli* were divided into 42 known STs, of which ST10 was predominant. In addition, we noticed that these isolates all carried multiple ARGs [6,7,8,9,10,11,12,13,14,15,16,17,18,19,20,21,22,23].

### 3.4. Genome Sequence Features of tet(X)-Positive E. coli

**Figure 2:**
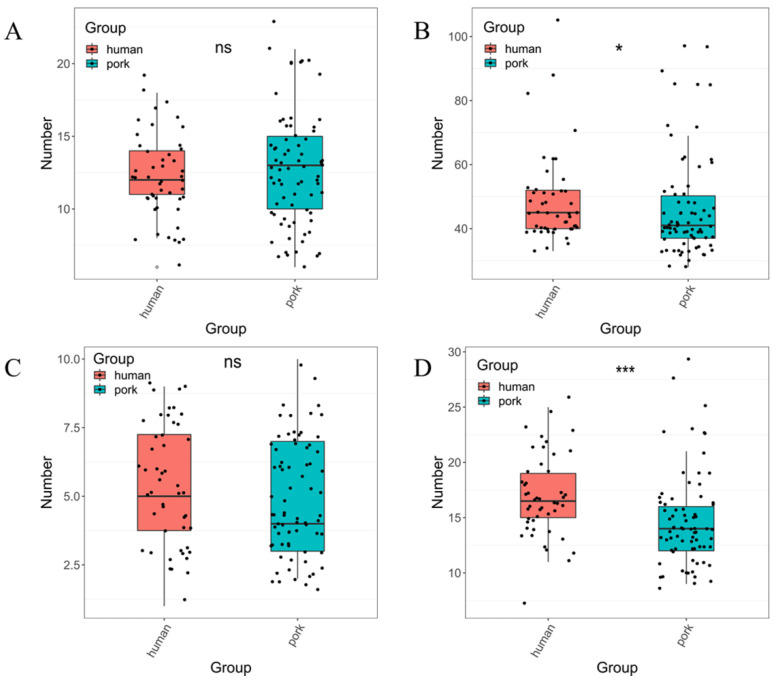
Genome analysis of 122 *tet*(X4)-positive *E. coli* collected from this study and NCBI database. (**A**) Number of ARGs carried by *E. coli* from different sources. (**B**) Number of virulence genes carried by *E. coli* from different sources. (**C**) Number of plasmid replicon types carried by *E. coli* from different sources. (**D**) Number of insertion sequences carried by *E. coli* from different sources. A dot represents an isolate. *: *p* < 0.05; ***: *p* < 0.001; ns: *p* > 0.05.

In order to further elucidate the genomic characteristics of *tet*(X4)-positive *E. coli* isolated from pork and humans, we counted the ARGs, virulence genes, plasmid replicons and insertion sequences carried by these *E. coli* isolates. As shown in Figure 2, the number of ARGs carried by *E. coli* of human origin was close to that carried by *E. coli* of pork origin, with no significant difference (*p* > 0.5). Similar to the results of ARGs, there was also no significant difference in the number of plasmid replicons carried by *E. coli* from two different sources (*p* > 0.5). However, *E. coli* of a human source carries far more virulence genes (*p* < 0.5) and insertion sequences (*p* < 0.001) than *E. coli* of a pork source.

### 3.5. Phylogenetic Analysis of tet(X)-Positive K. pneumoniae

**Figure 3:**
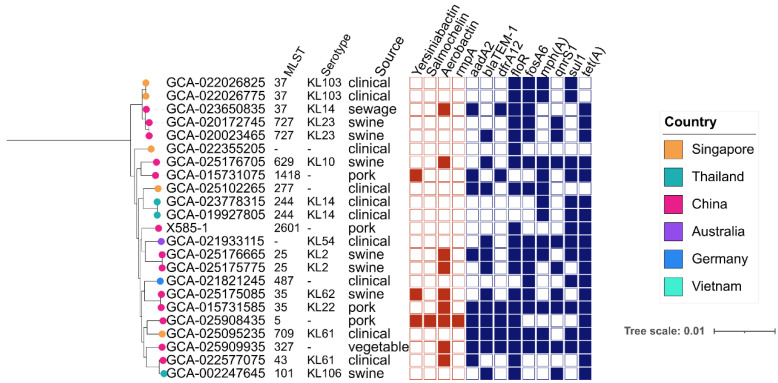
Phylogenetic relationship of 23 *tet*(X)-positive *K. pneumoniae* isolates. Resistance genes and virulence genes are indicated by squares; solid graphics indicate yes, and hollow graphics indicate no.

In addition to *E. coli*, a *tet*(X4)-positive *K. pneumoniae* isolate X585-1 was isolated in this study. We downloaded all *tet*(X)-positive *K. pneumoniae* (n = 29) from the NCBI database and constructed a phylogenetic tree based on SNPs of the core genomes (Figure 3, Table S5). We found that ST types and serotypes of the *tet*(X)-positive *K. pneumoniae* were diverse, and there were no dominant *tet*(X)-positive clones. These isolates were found in multiple countries but were mainly distributed in China (n = 18). Except for *tet*(X), these *K. pneumoniae* also carry multiple ARGs, including genes conferring resistance to β-lactams (*bla*_TEM-1_, n = 14), sulfonamides (*sul1*, n = 18), aminoglycosides (*aadA2*, n = 14), tetracyclines (*tetA*, n = 25) and trimethoprims (*drfA12*, n = 10). The *tet*(X)-positive *K. pneumoniae* carried only a small number of the virulence genes compared to the ARGs.

### 3.6. The Genetic Context of tet(X4) Carried by K. pneumoniae 

**Figure 4:**
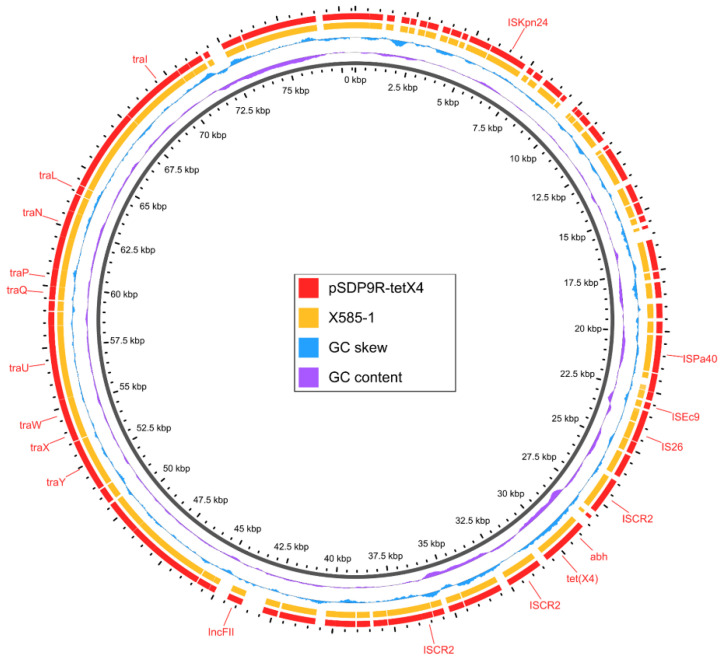
Circular comparison of the *tet*(X4)-bearing plasmid pSDP9R-tetX4 (NZ_MW940621) available in NCBI database and draft genome sequences of X585-1. The outermost circle with arrows denotes the reference plasmid pSDP9R-tetX4.

The BLAST comparison results indicated that the sequence of *K. pneumoniae* X585-1 exhibited high similarity to the online IncFII (pCRY) plasmid pSDP9R-tetX4 (NZ_MW940621) found in *K. pneumoniae* (Figure 4). This result implies that the *tet*(X4) gene was also located on the pSDP9R-tetX4-like plasmid. In addition to *tet*(X4), the *tet*(X4)-positive plasmid in X585-1 does not carry other ARGs. The core genetic environment of *tet*(X4) (IS*CR2*-*abh*-*tet*(X4)-IS*CR2*) carried by plasmid pMX581-tetX was the same as the plasmid pSDP9R-tetX4.

## 4. Discussion

Our previous investigation suggests that pork is an important reservoir of the *tet*(X4) gene [10]. However, there is still a lack of research on whether the *tet*(X4) gene carried in pork can spread to humans and the genome differences between *tet*(X4)-positive *E. coli* of human and pork origins. In this study, we use genomics to answer the above questions and provide some theoretical basis for subsequent research. A total of 23 *tet*(X4)-positive isolates were isolated from 53 pork samples, mainly *E. coli*, demonstrating that *E. coli* is an important host of *tet*(X4) among pork samples, which is consistent with the previous study [9]. The *tet*(X4) gene is usually located on different plasmid Inc types and can spread to the same or different bacterial species [8]. The *tet*(X4) gene isolated from pork samples was mainly located on the IncX1-IncHI2 and IncX1 plasmids. In addition, the IncX1 plasmid carrying *tet*(X4) usually has no significant fitness cost to the host, suggesting that the IncX1 plasmid is an important vector of the *tet*(X4) gene [10]. More than half of these *tet*(X4) genes were able to be successfully transferred into C600, indicating that these *tet*(X4) genes are located on mobile elements, such as plasmids. Most of these transferable plasmids were IncX1-type plasmids, highlighting that this type of plasmid may be more easily transferable to other strains [21].

Although the *tet*(X4) gene is mainly present in animal-derived samples, it has also been detected in human clinics in recent years [19]. Comprehensive genomic analysis proved that there is a possibility of clonal transmission of *tet*(X4)-positive isolates between pork samples and clinical samples. This phenomenon will greatly limit the choice of clinical medication and pose great challenges to public health. We noticed that these *tet*(X4)-positive *E. coli* isolated from pork and clinical samples all belonged to MDR isolates and carried a variety of ARGs. However, there was no significant difference in the number of ARGs carried by these two different sources of *E. coli*. In addition, we found that clinical samples carried significantly more virulence genes than pork samples. *E. coli* isolated from clinical samples carry more mobile elements. Mobile elements such as IS*CR2* and IS*26* play an important role in the spread and transfer of *tet*(X4), further exacerbating the spread of *tet*(X4) between different pathogens [23,24].

At present, *K. pneumoniae* has become the most important pathogen of nosocomial infections in China [25]. Some *K. pneumoniae*-evolved carbapenem-resistant *K. pneumoniae* and carbapenem-resistant hypervirulent *K. pneumoniae* have emerged, and tigecycline is regarded as the last choice for clinical treatment [26]. Although only a small number of *tet*(X)-positive *K. pneumoniae* are currently detected [12], they are detected in animal, environmental, as well as human-derived samples and require global vigilance. In addition, we found that *tet*(X)-positive *K. pneumoniae* had no dominant clones, indicating that mobile elements such as plasmids as well as insertion sequences play a key role in the spread of *tet*(X) genes. In addition to the *tet*(X) gene, we found that these *K. pneumoniae* also carry multiple ARGs, which are at risk of co-transmission. This phenomenon suggests that we need to revisit the importance of mobile elements in mediating the spread of ARGs.

## 5. Conclusions

In conclusion, *tet*(X4)-positive *E. coli* and *K. pneumoniae* in pork samples were systematically analyzed in this study. *tet*(X4)-positive *E. coli* isolates in pork samples were all MDR isolates. There is a possibility of the clonal transmission of *tet*(X4)-positive isolates between pork samples, as well as between pork and clinical samples. Notably, mobile elements may play a key role in the spread of *tet*(X) genes, which suggests that we should pay more attention to the role of these mobile genetic elements in the spread of ARGs.

## Data Availability

Not applicable.

## Supplementary Materials

The following are available online at https://www.mdpi.com/article/10.3390/genes14010036/s1, Table S1: The primers for detecting different plasmid replicons, Table S2: Antimicrobial susceptibility testing (MICs, mg/L) of 23 *tet*(X4)-positive strains, Table S3: Plasmid replicons of transconjugants, Table S4: Basic information of 117 *tet*(X4)-positive *E. coli* collected from the NCBI database, Table S5: Basic information of 22 *tet*(X)-positive *K. pneumoniae* genomes collected from the NCBI database.
