# Supplementary material for: Antimicrobial Resistance and Genomic Epidemiology of tet(X4)-Bearing Bacteria of Pork Origin in Jiangsu, China
Source: Genes (Basel). 2022 Dec 22;14(1):36. doi: 10.3390/genes14010036 (PMC9858217; doi:10.3390/genes14010036)
Supplement: Supplementary file 1 [file genes-14-00036-s001.zip › genes-2061949-supplementary.pdf]

Supplementary data

Table S1 The primers for detecting different plasmid replicons.

Primers	Sequences of 5'-3'	Products (bp)	Annealing temperature	Extension time
IncHI2A - F	GCTCGGGATCATCATGGCTT	175	52℃	30 s
IncHI2A - R	TGAGAAGGCAAGCACTACCC			
IncHI2 - F	AACACCCGGTTTCTACGCTT	218	53℃	30 s
IncHI2 - R	TAAGTTCCGGTGTCCTCCCA			
IncX1 - F	TGCCAGGGAGCGTAAGAAAC	177	51℃	30 s
IncX1 - R	ATACCACCCAGCCAGAACAC			
IncHI1B - F	TTACCGTCGACGCGATACAG	162	52℃	30 s
IncHI1B - R	AGCCCGGACCAACATTTCAT			
IncFII - F	CCGTAAGGTGGCAAGGAACT	111	52℃	30 s
IncFII - R	TGGGCCCCGGTAATCTTTTC			

Table S2 Antimicrobial susceptibility testing (MICs, mg/L) of 23 *tet*(X4)-positive strains.

Strain ID	Species	Transconjugant	MLST	Plasmid replicon type	Antimicrobials[a]								
					MEM	CIP	CHL	FFC	COL	STR	CFP	TIG	TET
X587-2-1-R	*E.coli*	-	-	IncHI1B, IncFII, IncX1, IncHI2A	<0.25	<0.25	128	>128	<0.25	>128	0.5	16	>128
X581-1-1	*E.coli*	+	-	IncX1, IncHI2A	<0.25	<0.25	128	>128	<0.25	128	>128	64	>128
X581-3-1	*E.coli*	-	-	IncX1, IncHI2A	<0.25	1	128	128	<0.25	>128	1	128	>128
X589-1-1	*E.coli*	-	-	IncX1, IncHI2A	<0.25	1	128	>128	<0.25	>128	64	32	>128
X615-1	*E.coli*	+	-	IncX1, IncHI2A	<0.25	<0.25	128	>128	<0.25	64	>128	64	>128
L586-3-1	*E.coli*	+	-	IncX1, IncHI2A	<0.25	<0.25	>128	>128	<0.25	>128	128	64	>128
X572-2-1-1	*E.coli*	-	-	IncX1, IncHI2A	<0.25	<0.25	>128	128	<0.25	64	64	128	>128
X572-2-2	*E.coli*	+	-	IncX1, IncHI2A	<0.25	<0.25	128	128	<0.25	>128	>128	32	>128
XX591-1	*E.coli*	+	-	IncX1, IncHI2A	<0.25	<0.25	>128	>128	<0.25	>128	<0.25	32	>128
X587-2-2-1-R	*E.coli*	-	-	IncHI1B, IncFII, IncX1, IncHI2A	<0.25	<0.25	128	128	<0.25	>128	0.5	32	128
X607-2-1	*E.coli*	-	-	IncX1, IncHI2A	<0.25	<0.25	64	128	<0.25	>128	<0.25	16	>128
X574-2-1-2	*E.coli*	+	-	IncX1, IncHI2A	<0.25	<0.25	128	>128	<0.25	64	>128	32	>128
L578-3-1	*E.coli*	+	2728	IncHI1B, IncHI2A	<0.25	<0.25	128	>128	<0.25	>128	0.5	32	>128
X594-2-1-2	*E.coli*	+	-	IncHI1B, IncFII, IncX1, IncHI2A	<0.25	<0.25	128	>128	<0.25	>128	>128	64	>128
X572-3-1	*E.coli*	+	-	IncX1, IncHI2A	<0.25	<0.25	128	>128	<0.25	>128	>128	64	>128
Lx600-2	*E.coli*	-	2064	IncHI1B, IncX1, IncHI2A	<0.25	4	>128	>128	0.5	8	2	128	>128
L584-2-1	*E.coli*	+	1725	IncHI1B, IncHI2, IncX1, IncHI2A	<0.25	<0.25	128	>128	<0.25	>128	0.5	128	>128
X591-3	*E.coli*	-	-	IncHI1B, IncX1, IncHI2A	<0.25	<0.25	128	>128	<0.25	>128	<0.25	64	>128
X573-1	*E.coli*	-	-	IncHI1B, IncFII, IncHI2A	<0.25	2	128	>128	<0.25	>128	<0.25	16	>128
X591-3-1-R	*E.coli*	+	-	IncHI1B, IncX1, IncHI2A	<0.25	4	>128	>128	<0.25	>128	64	32	>128
X594-2-1-1	*E.coli*	+	-	IncHI1B, IncX1, IncHI2A	<0.25	<0.25	64	128	<0.25	>128	<0.25	16	>128
L614-1	*E.coli*	+	-	IncX1, IncHI2A	<0.25	<0.25	>128	>128	<0.25	>128	64	32	>128
X585-1	*K. pneumoniae*	+	2601	IncFII	<0.25	>128	>128	>128	<0.25	>128	>128	8	64

[a] Abbreviations: MEM, meropenem; CIP, Ciprofloxacin; CHL, Chloramphenicol; FFC, florfenicol; COL, Colistin; STR, Streptomycin; CFP, Cefoperazone; TIG, tigecycline; TET, tetracycline.

Table S3 Plasmid replicons of transconjugants.

Transconjugant ID	Plasmid replicon type
CL572-3-1	IncX1
CL578-3-1	IncHI2
CX574-2-1-2	IncX1
CX585-1-1	IncFII
CX591-1	IncHI2A, IncX1
CL584-2-1	IncHI2A, IncX1
CX614-1	IncHI2A, IncX1
CX591-3-1-R	IncHI1B, IncHI2A
CX615-1	IncHI2A, IncX1
CX594-2-1-2	IncHI1B, IncHI2A, IncX1
CX586-3-1	IncHI2A, IncX1
CX572-2-2	IncHI2
CX594-2-1-1	IncHI1B, IncHI2A, IncX1
CX581-1-1	IncX1

Table S4 Basic information of 117 *tet*(X4)-positive *E. coli* collected from the NCBI database.

Strain	Isolation type	Isolation source	BioSample	Assembly
S555-1	clinical	Homo sapiens	SAMN19575535	GCA_019879125.1
S909-1	clinical	Homo sapiens	SAMN19575546	GCA_019879155.1
S985	clinical	Homo sapiens	SAMN19575549	GCA_019879205.1
S959	clinical	Homo sapiens	SAMN19575548	GCA_019879145.1
S557	clinical	Homo sapiens	SAMN19575536	GCA_019879245.1
S752	clinical	Homo sapiens	SAMN19575542	GCA_019879265.1
S934	clinical	Homo sapiens	SAMN19575547	GCA_019879285.1
S698-1	clinical	Homo sapiens	SAMN19575540	GCA_019879295.1
S542-2	clinical	Homo sapiens	SAMN19575533	GCA_019879345.1
S540-1	clinical	Homo sapiens	SAMN19575531	GCA_019879385.1
S518-1	clinical	Homo sapiens	SAMN19575530	GCA_019879405.1
S502-1	clinical	Homo sapiens	SAMN19575529	GCA_019879445.1
S455	clinical	Homo sapiens	SAMN19575525	GCA_019879485.1
S424-2	clinical	Homo sapiens	SAMN19575524	GCA_019879495.1
S383	clinical	Homo sapiens	SAMN19575522	GCA_019879525.1
S408-1	clinical	Homo sapiens	SAMN19575523	GCA_019879545.1
S364	clinical	Homo sapiens	SAMN19575521	GCA_019879535.1
S187-1	clinical	Homo sapiens	SAMN19575512	GCA_019879585.1
S314-1	clinical	Homo sapiens	SAMN19575519	GCA_019879605.1
S121-6	clinical	Homo sapiens	SAMN19575506	GCA_019879625.1
S274-2	clinical	Homo sapiens	SAMN19575518	GCA_019879645.1
S251-1	clinical	Homo sapiens	SAMN19575517	GCA_019879705.1
S221-6	clinical	Homo sapiens	SAMN19575515	GCA_019879735.1
S186-6	clinical	Homo sapiens	SAMN19575511	GCA_019879765.1
S180-1	clinical	Homo sapiens	SAMN19575510	GCA_019879785.1
S138	clinical	Homo sapiens	SAMN19575508	GCA_019879805.1
S107-2	clinical	Homo sapiens	SAMN19575504	GCA_019879845.1
S568-2	clinical	Homo sapiens	SAMN19575538	GCA_019879945.1
S35-1	clinical	Homo sapiens	SAMN19575520	GCA_019879965.1
S568-1	clinical	Homo sapiens	SAMN19575537	GCA_019879955.1
S855-1	clinical	Homo sapiens	SAMN19575544	GCA_019880005.1
R133-2	clinical	Homo sapiens	SAMN19575502	GCA_019880045.1
S90-6	clinical	Homo sapiens	SAMN19575545	GCA_019880065.1
R136-3	clinical	Homo sapiens	SAMN19575503	GCA_019880095.1
S847-1	clinical	Homo sapiens	SAMN19575543	GCA_019879885.1
S472	clinical	Homo sapiens	SAMN19575527	GCA_019879925.1
S110-1	clinical	Homo sapiens	SAMN19575505	GCA_019879685.1
S647	clinical	Homo sapiens	SAMN19575539	GCA_019879905.1
S544	clinical	Homo sapiens	SAMN19575534	GCA_019879225.1
S542	clinical	Homo sapiens	SAMN19575532	GCA_019879365.1
S221-2	clinical	Homo sapiens	SAMN19575514	GCA_019879635.1
S209-1	clinical	Homo sapiens	SAMN19575513	GCA_019879465.1
S229-2	clinical	Homo sapiens	SAMN19575516	GCA_019879725.1
S490-1	clinical	Homo sapiens	SAMN19575528	GCA_019879325.1
S468	clinical	Homo sapiens	SAMN19575526	GCA_019879425.1
S161-1	clinical	Homo sapiens	SAMN19575509	GCA_019879825.1
S130-6	clinical	Homo sapiens	SAMN19575507	GCA_019879855.1
S71-6	clinical	Homo sapiens	SAMN19575541	GCA_019880025.1
YPE10	environmental/other	pork	SAMN12233488	GCA_008807135.1
SZ12R	environmental/other	pork	SAMN16274518	GCA_015730745.1
SZ11R	environmental/other	pork	SAMN16274517	GCA_015730755.1
SX5G	environmental/other	pork	SAMN16274514	GCA_015730805.1
SX8G	environmental/other	pork	SAMN16274515	GCA_015730815.1
SZ10R	environmental/other	pork	SAMN16274516	GCA_015730825.1
SX3G	environmental/other	pork	SAMN16274513	GCA_015730835.1
SX2G	environmental/other	pork	SAMN16274512	GCA_015730885.1
SH6R	environmental/other	pork	SAMN16274507	GCA_015730925.1
SX13G	environmental/other	pork	SAMN16274510	GCA_015730935.1
SX1G	environmental/other	pork	SAMN16274511	GCA_015730955.1
SX11G	environmental/other	pork	SAMN16274509	GCA_015730945.1
SH11R	environmental/other	pork	SAMN16274503	GCA_015731015.1
SDP2R	environmental/other	pork	SAMN16274501	GCA_015731105.1
HS2-1	environmental/other	pork	SAMN16274494	GCA_015731245.1
HS17-1	environmental/other	pork	SAMN16274490	GCA_015731305.1
HS16-1	environmental/other	pork	SAMN16274489	GCA_015731345.1

HS12-1	environmental/other	pork	SAMN16274485	GCA_015731365.1
HS13-1	environmental/other	pork	SAMN16274486	GCA_015731415.1
HN13R	environmental/other	pork	SAMN16274480	GCA_015731515.1
HN16R	environmental/other	pork	SAMN16274481	GCA_015731505.1
AB4-2	environmental/other	pork	SAMN16274476	GCA_015731595.1
AB12-1	environmental/other	pork	SAMN16274472	GCA_015731705.1
2GS3	environmental/other	pork	SAMN16274470	GCA_015731725.1
ST20192942	environmental/other	pork	SAMN14119817	GCA_018295865.1
SRS6162984	environmental/other	pork	SAMN14119815	GCA_018295825.1
ST20192741	environmental/other	pork	SAMN14119813	GCA_018295765.1
ST20192836	environmental/other	pork	SAMN14119830	GCA_018295605.1
ST20192677	environmental/other	pork	SAMN14119829	GCA_018295625.1
ST20192631	environmental/other	pork	SAMN14119810	GCA_018295645.1
ST20193129	environmental/other	pork	SAMN14119828	GCA_018295585.1
SRS6162972	environmental/other	pork	SAMN14119827	GCA_018295505.1
ST20192618	environmental/other	pork	SAMN14119809	GCA_018295385.1
ST20192610	environmental/other	pork	SAMN14119797	GCA_018295325.1
YPE12	environmental/other	Pork	SAMN12233489	GCA_008807155.1
YPE3	environmental/other	Pork	SAMN12233487	GCA_008807295.1
SZ5R	environmental/other	pork	SAMN16274523	GCA_015730615.1
SH9W	environmental/other	pork	SAMN16274508	GCA_015730905.1
SH3W	environmental/other	pork	SAMN16274506	GCA_015731045.1
HS18-1	environmental/other	pork	SAMN16274491	GCA_015731265.1
ST20193088	environmental/other	pork	SAMN14119826	GCA_018295565.1
SZ7R	environmental/other	pork	SAMN16274525	GCA_015730605.1
HN18R	environmental/other	pork	SAMN16274483	GCA_015731455.1
HN17R	environmental/other	pork	SAMN16274482	GCA_015731485.1
HN10R	environmental/other	pork	SAMN16274479	GCA_015731545.1
AB5-1	environmental/other	pork	SAMN16274478	GCA_015731555.1
ZQ3-1	environmental/other	pork	SAMN16274527	GCA_015730585.1
HS9-1	environmental/other	pork	SAMN16274499	GCA_015731145.1
HS3-1	environmental/other	pork	SAMN16274495	GCA_015731165.1
HS6-1	environmental/other	pork	SAMN16274498	GCA_015731185.1
SZ12W	environmental/other	pork	SAMN16274519	GCA_015730705.1
AB3-1-R	environmental/other	pork	SAMN16274475	GCA_015731625.1
AB3-1-1	environmental/other	pork	SAMN16274474	GCA_015731645.1
AB1-1-1	environmental/other	pork	SAMN16274471	GCA_015731685.1
HS15-1	environmental/other	pork	SAMN16274487	GCA_015731405.1
HS10-1	environmental/other	pork	SAMN16274484	GCA_015731445.1
AB12-3	environmental/other	pork	SAMN16274473	GCA_015731655.1
SZ9R	environmental/other	pork	SAMN16274526	GCA_015730625.1
SZ3R	environmental/other	pork	SAMN16274522	GCA_015730685.1
SZ1R	environmental/other	pork	SAMN16274520	GCA_015730725.1
SZ2R	environmental/other	pork	SAMN16274521	GCA_015730715.1
HS5-1	environmental/other	pork	SAMN16274497	GCA_015731195.1
HS19-1	environmental/other	pork	SAMN16274492	GCA_015731315.1
SH13R	environmental/other	pork	SAMN16274505	GCA_015731005.1
SH12R	environmental/other	pork	SAMN16274504	GCA_015731065.1
HS4-1	environmental/other	pork	SAMN16274496	GCA_015731225.1
HS15-2	environmental/other	pork	SAMN16274488	GCA_015731385.1
SRS6162985	environmental/other	pork	SAMN14119816	GCA_018295805.1
SRS6162983	environmental/other	pork	SAMN14119814	GCA_018295785.1

Table S5. Basic information of 22 *tet*(X)-positive *K. pneumoniae* genomes collected from the NCBI database.

Strain	Location	Isolation source	Isolation type	Host	BioSample	Assembly No.
MRSN581745	Thailand	urine	clinical	Homo sapiens	SAMN18874753	GCA_019927805.1
Microbial	Thailand: Chonburi	urine	clinical	Homo sapiens	SAMN28592075	GCA_023778315.1
2016CRE_8	Singapore	Intermediate-Care Facility	clinical	Homo sapiens	SAMN16824633	GCA_022026825.1
2016CRE_7	Singapore	Intermediate-Care Facility	clinical	Homo sapiens	SAMN16824632	GCA_022026775.1
K-1L	China:Guangxi	NA	NA	swine	SAMN18487815	GCA_020023465.1
b1-2L	China:Guangxi	NA	NA	swine	SAMN18487814	GCA_020172745.1
AUSMDU00041478	Australia	feces	clinical	Homo sapiens	SAMN20033638	GCA_021933115.1
SDP9R	China	pork	environmental/other	pork	SAMN16274502	GCA_015731075.1
AB4-4	China	pork	environmental/other	pork	SAMN16274477	GCA_015731585.1
210-ESBLB	Singapore	rectal swab	clinical	Homo sapiens	SAMN09460085	GCA_022355205.1
T877	China:Guangdong	NA	clinical	Homo sapiens	SAMN21849140	GCA_022577075.1
GD21SC417	China:Guangdong	NA	NA	bacteria	SAMN23799605	GCA_023650835.1
S.15.20.Kp	Germany	hospital	clinical	Homo sapiens	SAMEA8218291	GCA_021821245.1
TQ11	China	fresh feces	NA	swine	SAMN27625142	GCA_025175085.1
TQ30	China	fresh feces	NA	swine	SAMN27625141	GCA_025175775.1
JZ18	China	fresh feces	NA	swine	SAMN27625117	GCA_025176665.1
JZ47	China	fresh feces	NA	swine	SAMN27625115	GCA_025176705.1
EC3266	Singapore	wound	clinical	Homo sapiens	SAMN17981844	GCA_025095235.1
EC1807	Singapore	URINARY	clinical	Homo sapiens	SAMN17981743	GCA_025102265.1
GD21SC1725T	China:Guangdong	vegetable	NA	Cantonese cabbage	SAMN31416123	GCA_025909935.1
YZ-58	China	pork	environmental/other	pork	SAMN28157411	GCA_025908435.1
KPSW+02	Thailand: Lamphun	Rectal swab	clinical	Swine	SAMN07450639	GCA_002247645.1

NA, not available.
